# ACTIVOT: PRELIMINARY RESULTS OF AN INTERVENTION TO IMPROVE HEALTH-PROMOTING DAILY ACTIVITY

**DOI:** 10.1093/geroni/igae098.2848

**Published:** 2024-12-31

**Authors:** Tara Klinedinst, Nicholas Hollman, Carrie Ciro, Darla Kendzor

**Affiliations:** University of Oklahoma Health Sciences Center, Oklahoma City, Oklahoma, United States; OU-TU School of Community Medicine, Tulsa, Oklahoma, United States; University of Oklahoma Health Sciences Center, Oklahoma City, Oklahoma, United States; University of Oklahoma Health Sciences Center, Oklahoma City, Oklahoma, United States

## Abstract

Nearly half of older adults in the U.S. have multiple chronic conditions in addition to functional limitations that restrict health-promoting daily activity (e.g., sleep, medication-taking, physical activity). We tested an innovative combination of behavioral activation and occupational therapy techniques to improve performance of meaningful, healthy activity. The ActivOT intervention was delivered by occupational therapists in 10 weekly sessions in participant homes. The primary outcome is performance of health-promoting daily activity, measured by the Canadian Occupational Performance Measure (COPM) at baseline, 10-weeks, and 22-weeks. We report outcome data from 17 participants who were randomized to ActivOT or brief education (n=9 Tx, n=8 Control). On average, participants were 73.0 years old (SD=6.9), majority female (n=13), and White (n=1 Black, n=1 Hispanic/Latino). They had an average of 3.9 chronic conditions (SD=1.4), and 1.9 functional limitations (SD=1.7). The average COPM performance score (out of 10) at baseline was similar for the ActivOT group (M=3.6, SD=1.6) and control group (M=3.3, SD=1.1). At 10-week follow-up, the ActivOT group average increased to 7.0 (SD=1.7) and further increased to 7.2 (SD=1.0) at 22-weeks. In comparison, the control group average increased to 5.9 (SD=2.0) at 10-weeks, then decreased to 5.0 (SD=2.2) at 22-weeks. Further, 89% percent of the ActivOT group reported clinically significant improvements on the COPM (MCID=3). Results show an initial increase in both groups, but the increases are only maintained in the ActivOT group. This study is in progress (aiming for n=40) but shows promise for improving performance of health-promoting daily activity in a difficult-to-treat population.

